# Psychological impact of COVID19 on community pharmacists and pharmacy technicians

**DOI:** 10.1016/j.rcsop.2022.100118

**Published:** 2022-03-01

**Authors:** Carmen Baldonedo-Mosteiro, Sara Franco-Correia, Maria-Pilar Mosteiro-Diaz

**Affiliations:** a University of Oviedo. Campus el Cristo s/n, 33006 Oviedo, Spain; b Faculty of Medicine and Health Sciences. University of Oviedo, Campus el Cristo s/n, 33006 Oviedo, Spain

**Keywords:** Pharmacists, Pharmacy technicians, Pandemic, COVID-19, Lockdown

## Abstract

**Background:**

The unique situation related to the COVID-19 outbreak and the consequent worldwide lockdown can have a psychological impact on specific populations. Community pharmacists and pharmacy technicians, as essential healthcare workers on the front line who continue to do their jobs during this pandemic, can also experience psychological distress. Few data are available on the mental health impact of the COVID-19 pandemics on this population.

**Objectives:**

This study aimed to assess the psychological impact of COVID-19 on Spanish community pharmacists and pharmacy technicians during lockdown, and to identify factors contributing to psychological distress.

**Methods:**

A cross-sectional, quantitative, correlational study was designed including 1162 pharmacy team members. The Impact of Event Scale revised was used to assess the psychological impact. Data collection was performed by emailing the instrument to individuals or by using social networks.

**Results:**

Overall participants, almost 70% revealed severe levels of psychological impact. The outbreak of COVID-19 has significantly affected community pharmacy workers, the degree of which is related to gender, age, and feelings of fear/stress.

**Conclusions:**

In the initial phase of the lockdown associated with the COVID-19 outbreak, the majority of the respondents rated the psychological impact as severe. Our findings allow for the identification of factors associated with a greater psychological impact.

## Highlights

• Community pharmacists and pharmacy technicians reveal severe levels on IES-R.
• Higher levels of psychological impact were associated to gender, age, feeling stress and fear.
• Local incidence and pharmacy location seem to be related to severe levels on IES-R.

## 1 Introduction

On 31st December 2019, the World Health Organization (WHO) was advised by the National Health Commission in China of an outbreak of 27 cases of pneumonia caused by a new coronavirus.^1^ The new virus was later officially named severe acute respiratory syndrome coronavirus (SARS-COV-2)^2^ and was first detected in Wuhan, China, associated with exposure in a seafood market.

The coronavirus disease (COVID-19) caused by SARS-COV-2 is a highly infectious disease that can lead to serious complications such as acute respiratory distress syndrome, acute renal failure, septic shock and ventilator-associated pneumonia.^2^ Due to the wide spread of the virus, the Spanish government declared a state of emergency. This resulted in strict isolation, requiring people to stay at home and imposing the closure of all non-essential businesses. As essential businesses, community pharmacies played an important role in the prevention of the spread of the COVID-19 outbreak and helped with overall emergency management.^3^

The crucial role pharmacists play in the provision of health care has been highlighted by the pandemic.^4^ All around the world during the pandemic, pharmacists have been integrated into planning and responses.^5^^,^^6^ Facing public health emergencies like a pandemic, pharmacists' activity is based on taking advantage of their pharmacological expertise to participate actively in the medical activities related to COVID-19, and to maximize pharmacists' value and responsibility.^7^

During the pandemic, pharmacists, and especially, community pharmacists, are considered by the population as a valuable resource. In community settings, they can play an important role by enhancing health awareness among the public and also by making pharmaceutical products available.^8^

In some rural areas during the lockdown, while health centres and local clinics were closed and many hospital appointments were cancelled, pharmacies became the only health point accessible to all. In addition to their usual responsibilities, pharmacists had to collaborate with government, in activities such as maintaining a stable supply of medication and hygiene products. ^3^ They are, therefore, considered frontline healthcare staff uniquely placed to provide healthcare to a large portion of the population and have excellent potential to contribute to the pandemic response.^9^ While the local government was asking the country to stay at home, pharmacists and pharmacy technicians had to keep going to work every day, knowing there was not enough personal protective equipment (PPE) to face the virus. Pharmacists and technicians could be vulnerable to mental health problems and may have feared spreading the infection to their family members, friends, or colleagues.

Globally the COVID-19 outbreak has influenced the mental health of healthcare staff.^10^ Stress, anxiety, depressive symptoms, and exacerbations of pre-existing mental illness may be increased due to the challenging conditions imposed.^11^, ^12^, ^13^, ^14^ Pharmacists, as the most accessible healthcare staff, may face many stressors that need to be dealt with in order to effectively address pharmacist's mental health.^9^ Few data are available on the mental health impact of the COVID-19 pandemic in this population in Spain. The following research question was defined: ‘What is the psychological impact of COVID-19 outbreak lockdown on pharmacy staff in Spain?’ The aim of this study was to assess the psychological impact of the COVID-19 on community pharmacists and pharmacy technicians during the lockdown in Spain, and to identify factors contributing to psychological distress.

## 2 Material and methods

### 2.1 Design, sample and setting

A cross-sectional, correlational, quantitative study was performed. Data were collected from pharmacists and pharmacy technicians during the COVID-19 outbreak from 4 April to 21 April 2020. A convenient snowball sampling method was used (*N* = 1162). Pharmacy workers were sent an e-mail with information about the study and a link to the questionnaire. The same information was disseminated through social networks. Prior to instrument application, a consent form was presented to all participants. By answering the data collection instrument, participants provided consent for their data to be used in the study. No incentive was provided for survey completion.

### 2.2 Measurement tool

The Impact of Event Scale Revised (IES-R) Spanish version^15^ was used in this study. The IES-R^16^ has been applied as a self-report measure to assess the level of symptomatic response to specific traumatic events as it was manifested in the previous 7 days. It consists of a brief self-administered 22-item questionnaire, and for response uses a five-point Likert scale. Scale scoring of IES-R includes a total score (ranging from 0 to 88) and three subscales reflecting intrusion (8 items), avoidance (8 items), and hyperarousal (6 items) symptoms.^17^ The IES-R is considered an appropriate instrument to measure the subjective response due to a specific traumatic event, as COVID-19 pandemics. It allows to divide the symptoms into 3 subscales: Intrusion, avoidance and hyperarousal. Intrusion symptoms are intrusive thoughts, nightmares, intrusive feelings and imagery, dissociative-like re-experiencing. Numbing of responsiveness, avoidance of feelings, situations, and ideas are considered avoidance symptoms. In turn, hyperarousal symptoms include anger, irritability, hypervigilance, difficulty concentrating, heightened startle. The total score indicates the global subjective stress regarding to the identified event.^18^

Higher levels of distress are reflected by higher total (or subscale) scores.^18^ The total IES-R score was divided into normal (0−23), mild psychological impact (24–32), moderate psychological impact (33–36), and severe psychological impact (≥37).^19^

Sociodemographic data such as gender, age, marital status, children, coexistence, dependents, professional category, geographic region, smoking habits, pharmacy location, and local incidence of COVID-19 were collected. Local incidence is here defined as the regional incidence of COVID cases in the survey (stratified into different classes). Independent variables, assessed by simple questions with dichotomous answer (yes/no) were added: ‘Do you feel fear?’; ‘Do you feel stress?’; ‘Were you infected?’; ‘Do you have any infected close family members?’; ‘Do you have any infected friends?’, ‘Do you think pharmacists/pharmacy technicians are very exposed?’, and ‘Do you live with anyone who has been infected?’

### 2.3 Procedure

A digital form was sent to pharmacy staff all around the country after obtaining approval from the ethical committee - Ethics Committee for Investigation of the Principality of Asturias (2020.116). Prior to completing the questionnaire, basic information about the study was provided to participants. A guarantee of confidentiality and anonymity in relation to data was ensured. After reading both basic information and confidentiality and anonymous aspects, they we asked to mark an agree consent box before they had access to the questionnaire.

### 2.4 Statistical analyses

The SPSS program, version 24 was used for data analyses. A descriptive analysis of each collected variable was performed, by mean, median, minimum, and maximum. Measures of dispersion such as the standard deviation was used to quantitative variables. For qualitative type variables absolute and relative frequency distributions was used. The Student *t-*test for independent samples was used to detect differences of quantitative variables in two groups, but before the compliance with the normality hypothesis was assessed. The ANOVA test or the Kruskal-Wallis test were applied for three or more groups, depending on whether or not the hypotheses of normality and/or homoscedasticity were verified.

## 3 Results

### 3.1 Descriptive statistics

The sample was formed by 1162 participants who fully completed the queries between 4 April to 21 April 2020. More than half (63.6%) were pharmacists, with a mean age of 39.15 ± 9.718 [20; 65]. Most were female (86.7%), married (67.2%), with no children (50.6%), non-smokers (72.9%), from the northern region of the country (45.2%), working in large city pharmacies (40.1%), with a local incidence of COVID-19 of 10,001–15,000 (27.5%). The majority (94.0%) thought that pharmacists/pharmacy tecnicians are very exposed. Within the participants, more than half self-reported feeling fear (55.9%) and a larger number reported feeling stress (90.4%) (see Table 1).

**Table 1:** Sample characteristics.

Variable	Type	Frequency	%
Gender	Female Male	1008	86.7
		154	13.3
Professional category	Pharmacist Pharmacy technician	739	63.6
		423	36.4
Marital status	Single Married/living with partner Divorced/separated Widow	312	26.9
		781	67.2
		61	5.2
		8	0.7
Geographic region	North Central South Islands Not recorded	525	45.2
		386	33.2
		167	14.6
		68	5.9
		16	1.4
Local incidence of COVID-19^a^	< 3000 3000–5000 5001–10,000 10,001–15,000 15,001–20,000 >20,000	295	25.4
		64	5.5
		90	7.7
		320	27.5
		130	11.2
		263	22.6
Pharmacy location^b^	Town Small city Large city	362	31.2
		333	28.7
		466	40.1
Smoking habits	No Tobacco *E*-cigarettes Ex-smoker	847	72.9
		203	17.5
		13	1.1
		99	8.5
Children	No Yes	588	50.6
		574	49.4
Do you think pharmacists/pharmacy technicians are very exposed?	No Yes	70	6.0
		1092	94.0
Do you feel fear?	No Yes	513	44.1
		649	55.9
Do you feel stress?	No Yes	111	9.6
		1051	90.4
Do you have any infected close family members?	No Yes	991	85.3
		171	14.7
Do you have any infected friends?	No Yes	709	61.0
		453	39.0
Do you live with anyone who has been infected?	No Yes	1106	95.2
		56	4.8

a Regional incidence of COVID-19 cases in the survey period.

b One participant did not respond to the pharmacy location question.

Participants presented mean scores on IES-R subscales higher than 14.5. Regarding to IES-R, the global score mean was 44.95. Almost 70% of the participants revealed a severe psychological impact (IES-*R* ≥ 37) (see Table 2).

**Table 2:** IES-R test results.

	Mean ± SD		[min -max]
SUBSCALE			
Intrusion	14.74 ± 7.084		0–28
Avoidance	15.65 ± 7.065		0–32
Hyperarousal	14.55 ± 6.943		0–28
IES-R global	44.95 ± 19.660		0–88

IES-R LEVELS	Total sample N (%)	Pharmacist N (%)	Pharmacy technician N (%)

Normal	201 (17.3)	134 (18.13)	67 (15.84)
Mild	118 (10.2)	75 (10.15)	43 (10.17)
Moderate	41 (3.5)	28 (3.79)	13 (3.07)
Severe	802 (69.0)	502 (67.93)	300 (70.92)

The results indicated that gender, age, and feeling fear or feeling stress seems to be associated with higher IES-R scores. Also, the local incidence of COVID-19, pharmacy location and ‘Think pharmacists/pharmacy technicians are very exposed’ were statistically related to severe levels on the IES-R (see Table 3). A correlational analysis dividing the sample in pharmacists and pharmacy technicians was performed (see Table 4).

**Table 3:** Variables statistically associated with IES-R levels.

Variable		EIS-R levels					p
		Normal	Mild	Moderate	Severe	Total	
Gender	Female Male	149 (12.82)	99 (8.52)	38 (3.27)	722 (62.13)	1008 (86.75)	<0.001
		52 (4.48)	19 (1.64)	3 (0.26)	80 (6.88)	154 (13.25)	
Local incidence of COVID-19	<3000 3001–5000 5001–10,000 10,001–15,000 15,001–20,000 >20,000	73 (6.28)	39 (3.36)	13 (1.12)	170 (14.63)	295 (25.39)	0.003
		13 (1.12)	8 (0.69)	2 (0.17)	41 (3.53)	64 (5.51)	
		9 (0.77)	9 (0.77)	3 (0.26)	69 (5.94)	90 (7.75)	
		51 (4.39)	28 (2.41)	13 (1.12)	228 (19.62)	320 (27.53)	
		24 (2.06)	12 (1.03)	4 (0.34)	90 (7.75)	130 (11.19)	
		31 (2.67)	22 (1.89)	6 (0.52)	204 (17.56)	263 (22.63)	
Pharmacy location	Town Small city Large city	59 (5.08)	38 (3.27)	9 (0.77)	256 (22.03)	362 (31.15))	0.039
		50 (4.30)	27 (2.32)	12 (1.03)	244 (21.00)	333 (28.66)	
		92 (7.92)	53(4.56)	20 (1.72)	302 (26.00)	467 (40.19)	
Do you think pharmacists/pharmacy technicians are very exposed?	No Yes	31 (2.67)	7 (0.60)	1 (0.09)	31 (2.67)	70 (6.02)	<0.001
		170 (14.63)	111 (9.55)	40 (3.44)	771 (66.35)	1092 (93.98)	
Do you feel fear?	No Yes	153 (13.17)	68 (5.85)	19 (1.64)	273 (23.49)	513 (44.15)	<0.001
		48 (4.13)	50 (4.30)	22 (1.89)	529 (45.53)	649 (55.85)	
Do you feel stress?	No Yes	69 (5.94)	20 (1.72)	2 (0.17)	20 (1.72)	111 (9.55)	<0.001
		132 (11.36)	98 (8.43)	39 (3.36)	782 (67.30)	1051 (90.45)	
Age	Mean (SD)	41.1(±10.8)	40.5(±9.5)	38.6(±9.4)	38.5(±9.4)	39.1(±9.7)	0.002

**Table 4:** Variables statistically associated with IES-R levels: pharmacists vs pharmacy technicians.

Variable			EIS-R LEVELS n (%)				p
	Professional category		Normal	Mild	Moderate	Severe	
Gender	Pharmacist	Female Male	96 (12.99)	59 (7.98)	26 (3.52)	443 (59.95)	<0.001
			38 (5.14)	16 (2.17)	2 (0.27)	59 (7.98)	
	Pharmacy technician	Female	53 (12.53)	40 (9.46)	12 (2.84)	279 (65.96)	0.005
		Male	14 (3.31)	3 (0.71)	1 (0.23)	21 (4.96)	
Local incidence of COVID-19	Pharmacy technician	<3000 3001–5000 5001–10,000 10,001–15,000 15,001–20,000 >20,000	27 (6.38)	15 (3.55)	4 (0.95)	60 (14.18)	0.027
			3 (0.71)	1 (0.24)	0	17 (4.02)	
			1 (0.24)	3 (0.71)	0	20 (4.73)	
			25 (5.91)	13 (3.07)	5 (1.18)	89 (21.04)	
			5 (1.18)	3 (0.71)	2 (0.47)	35 (8.27)	
			6 (1.42)	8 (1.89)	2 (0.47)	79 (18.68)	
Do you think pharmacists/pharmacy technicians are very exposed?	Pharmacist	No Yes	25 (3.38)	6 (0.81)	0	15 (2.03)	<0.001
			109 (14.75)	69 (9.34)	28 (3.79)	487 (65.9)	
Do you feel fear?	Pharmacist	No Yes	104 (14.07)	46 (6.23)	12 (1.62)	190 (25.71)	<0.001
			30 (4.06)	29 (3.92)	16 (2.17)	312 (42.22)	
	Pharmacy technician	No Yes	49 (11.58) 18 (4.26)	22 (5.20) 21 (4.97)	7 (1.65) 6 (1.42)	83 (19.62) 217 (51.30)	<0.001
Do you feel stress?	Pharmacist	No Yes No Yes	46 (6.22)	15 (2.03)	2 (0.27)	14 (1.89)	<0.001 <0.001
			88 (11.91)	60 (8.12)	26 (3.52)	488 (66.04)	
	Pharmacy technician		23 (5.44) 44 (10.40)	5 (1.18) 38 (8.98)	0 13 (3.08)	6 (1.42) 294 (69.50)	
Do you have any infected friends?	Pharmacy technician	No Yes	52 (12.29) 15 (3.55)	33 (7.80) 10 (2.36)	11 (2.60) 2 (0.47)	188 (44.44) 112 (26.47)	0.022

Using an ANOVA test analysis, it's possible to clarify that the flowing variables have an effect on global IES-R score: age, gender, “Do you think pharmacist/pharmacy technicians are very exposed?”, “Do you feel fear?”, “Do you feel stress?”, “Do you have any infected friend?” and Local incidence (see Table 5a). A parameter estimation allows us to identify that being a female increases on 4.65 the chances of having high IES-R scores, when compared to male. Also, answering “No” to the question “Do you think pharmacist/pharmacy technicians are very exposed?” it's associated to 7.112 chances to have lower IES-R scores, comparing to whom who answer “yes” to the question. Not feeling fear or stress are associated to high probabilities of having lower IES-R scores that those who feel stress or fear (see Table 5b).

**Table 5a:** ANOVA analysis.

		Sum of Squares	Mean square	F	Sig.
Corrected model		128,645.498^a^	16,080.687	57.920	0.000
Interception		41,774.314	41,774.314	150.465	0.000
Age		1781.971	1781.971	6.418	0.011
Gender		2765.783	2765.783	9.962	0.002
Professional category		166.239	166.239	0.599	0.439
Do you think pharmacist/pharmacy technicians are very exposed?		3202.339	3202.339	11.534	0.001
Do you feel fear?		32,044.378	32,044.378	115.419	0.000
Do you feel stress?		37,295.455	37,295.455	134.333	0.000
Do you have any infected friends?		2296.712	2296.712	8.272	0.004
Local incidence		4499.498	4499.498	16.207	0.000
Dependent Variable: EIS-R global score					

a R^2^ = 0.287 (Adjusted R^2^ = 0.282)

**Table 5b:** Parameter estimation.

Parameter	B	t	95% CI	
			Lower bound	Upper bound
Interception	50.690	17.583	45.034	56.346
Age	−0.131	−2.533	−0.233	−0.030
[Gender = female]	4.646	3.156	1.758	7.534
[Gender = male]	0^a^	.		
[Professional category = Pharmacist]	0.802	0.774	−1.232	2.837
[Professional category = pharmacy tecnichian]	0^a^			
[Do you think pharmacist/pharmacy technicians are very exposed? = No]	−7.112	−3.396	−11.220	−3.003
[Do you think pharmacist/pharmacy technicians are very exposed? = Yes]	0^a^			
[Do you feel fear? = No]	−11.054	−10.743	−13.072	−9.035
[Do you feel fear? = Yes]	0^a^			
[Do you feel stress? = No]	−20.126	−11.590	−23.533	−16.719
[Do you feel stress? = Yes]	0^a^			
[Do you have any infected friends? = No]	−2.954	−2.876	−4.969	−0.939
[Do you have any infected friends? = Yes]	0^a^			

a Parameter is set to 0 because it is redundant.

## 4 Discussion

This study, aimed to determine the psychological impact of the COVID-19 pandemic on community pharmacists and pharmacy technicians. A total sample of 1162 participants was obtained, which included both pharmacists and technicians. Searching out for other studies that could help to interpret the results, it was determined that there are not many works available. Despite the fact that there are few studies related to this topic in this specific population, it was found appropriate to compare and discuss the results based on studies on healthcare professionals as well as on general population surveys, wherever possible.

### 4.1 IES-R test results

When analysed the IES-R scores, a high incidence of severe levels of psychological impact was detected (*n* = 802). This included a large number of people, representing almost 70% of the total sample. In a French study also conducted during the COVID-19 outbreak, in a sample of 135 community pharmacists, twenty-three pharmacists (17%) reported significant post-traumatic stress symptoms also by using IES-R.^10^ In a study with 470 medical and non-medical healthcare workers, IES-R scores were higher in non-medical healthcare professionals (where pharmacists were included).^20^, ^21^ It is important to highlight the higher scores on IES-R present in pharmacists when compared to other population groups. Other studies in healthcare professionals^21^ or in students^22^ during the outbreak presented a lower incidence of severe levels. In China, in a survey on 1210 participants from the general public, 53.8% of respondents rated the psychological impact of the outbreak as moderate or severe.^23^ In contrast to our results, in another work, with a sample of 906 healthcare professionals from Singapore and India, the total IES-R mean score was 8.29 (SD 9.79).^21^ Despite the fact that no available evidence was found to explain the results in the current study, perhaps the proximity to hard realities experienced in very similar sociocultural contexts may have contribute to the high psychological impact generated in this population. It's important to clarify that many frontline care workers in close countries were dying because of COVID-19(eg: Italy).

### 4.2 Associated variables

Comparing IES-R global scores and gender, age, and feeling fear or feeling stress, statistical differences were detected, as these variables were found to be related to high levels on IES-R (*p* < 0.05). In fact, being women and younger seems to be a predictor to high IES-R scores.

When performed the same analyses by professional category – pharmacists and pharmacy technicians – similar association were detected in both groups regarding to high levels on IES-R and the following variables: gender, felling fear and felling stress. However, in the pharmacists group a statistical association was found between severe levels on IES-R and “Do you think pharmacists/pharmacy technicians are very exposed?”. In the other hand, an association was identified in the pharmacy technician group between higher levels on IES-R and local incidence of COVID-19.

Our study revealed that being female was associated with higher IES-R scores. In another study, gender was positively correlated with higher IES-R scores.^24^ Another study pointed out that female gender was significantly associated with a greater psychological impact of the outbreak.^25^ In addition, being female was found to be associated with higher scores than being male for IES-R (*p* = 0.01) in a French pharmacist sample.^10^ Similarly, in a study that compared the impact of the COVID-19 outbreak on 470 medical and non-medical participants, higher IES-R total and subscale scores were observed in non-medical healthcare workers.^24^ However, in the referred study, the overall mean of IES-R among healthcare workers was lower than in other published studies, including studies reflecting the psychological impact during SARS outbreak.

In terms of age, results showed that there was a relationship between high IES-R score and younger ages. In another study, performed on medical staff and the general public, the data indicated that age was positively correlated with IES-R^24^. It's possible that the obtained data are related due the fact that usually younger people adopt fewer coping strategies and present lower levels or resilience.

In addition, results suggest that those individuals who self-reported feeling subjective fear and stress revealed higher levels on the IES-R. Although no other studies were found comparing these two variables, a study on healthcare professionals during the MERS-CoV outbreak in Saudi Arabia indicates that the staff did feel fear.^24^ Also, Lima et al ^25^ stated that fear seems more certainly to be a consequence of mass quarantine.

Additionally, data indicate that the local incidence of COVID-19, the pharmacy location and ‘Do you think pharmacists/pharmacy technicians are very exposed?’ were associated with severe levels on IES-R. In fact, pharmacists working in large city pharmacies revealed levels considered severe on IES-R. Regarding local incidence of COVID-19, participants in areas with a local incidence of COVID-19 of 10,001–15,000 scored higher in IES-R. A large majority of participants (90.45%) who believe that ‘pharmacists are very exposed’ were those who presented higher psychological impact related to COVID-19. In a previous study on pharmacists and pharmacy students during the current pandemic, 90% of the participants stated being aware of their role counseling the public regarding COVID-19 infection. The study also highlighted the importance given to their personal safety avoiding close contacts.^8^ It's possible that this fact justifies the general sense of our sample, who had the sensation of being very exposed to the virus in their professional activity, as essential frontline workers. Also, Chen et al. found that working during the epidemic showed a positive correlation with IES-R.^22^ A lower psychological impact was associated with being accurately updated on health information and prevention measures (including local outbreak status or hand hygiene and masks).^21^ Regarding these last variables, little information was found on pharmacists that could help on finding's discussion.

These results should be interpreted in light of the numbers of infected and dead healthcare workers. In Spain, up to 25 June 2020, 52,575 confirmed cases of COVID-19 were noted in healthcare professionals.^26^

### 4.3 Limitations

As limitations to our study, the use of a convenient snowball sampling technique may have biased our results. By sending the instrument by email or using social networks, participants could feel less involved in the study. Also, the self-report nature of the survey and influence of socially acceptable answers could have been considering as a limitation. Not asking about previous history of mental illness can be considered itself a limitation.

## 5 Conclusions

To the authors knowledge this is the first study to show the psychological impact of COVID-19 in Spanish community pharmacists. The psychological impact on pharmacists during the COVID-19 pandemic was assessed with the IES-R, and severe levels were found in a large number of participants. Higher scores seem to be associated with some individual characteristics such as being female and young. In this study, participants that self-reported feeling stress and fear had severe levels on the IES-R. Also, pharmacy location, the local incidence of COVID-19 and ‘think that pharmacists/pharmacy technicians are very exposed’ were associated with higher scores on IES-R.

Further large studies are necessary to help us understand, explain and find strategies to support these professionals in reducing their levels of psychological impact related to the pandemic that we are all going through. It would also be interesting to compare pharmacy workers in different countries. In the other hand, on a pandemic with such consequences the implementation of global programmes dedicated to mental health in frontline healthcare professionals, including pharmacists and pharmacy technicians is needed. Policies regarding to prevent and to treat psychological impact on this population are urgent.

## Funding

This research received no specific grant from any funding agency in the public, commercial, or not-for-profit sectors.

## Data availability

The data underlying this article will be shared on reasonable request to the corresponding author.

## Declaration of Competing Interest

The authors declare that there are no conflicts of interest.
